# Time of Test Periods Influence the Behavioral Responses of *Anopheles minimus* and *Anopheles dirus* (Diptera: Culicidae) to DEET

**DOI:** 10.3390/insects12100867

**Published:** 2021-09-24

**Authors:** Rungarun Tisgratog, Chutipong Sukkanon, Victor Arief Sugiharto, Michael J. Bangs, Theeraphap Chareonviriyaphap

**Affiliations:** 1 Department of Entomology, Faculty of Agriculture, Kasetsart University, Bangkok 10900, Thailand; faasthc@ku.ac.th; 2 Department of Medical Technology, School of Allied Health Sciences, Walailak University, Tha Sala 80160, Thailand; chutipong.su@wu.ac.th; 3 Henry M Jackson Foundation for the Advancement of Military Medicine, Bethesda, MD 20817, USA; va.sugiharto@gmail.com; 4 Public Health & Malaria Control Department, PT. Freeport Indonesia, International SOS, Kuala Kencana, Mimika Regency 99920, Indonesia; bangs_michael@yahoo.com

**Keywords:** *Anopheles minimus*, *Anopheles dirus*, time of test, avoidance behavioral response, DEET

## Abstract

**Simple Summary:**

The influence of environmental and physiological factors on various aspects of normal mosquito behavior is unclear. The efficacy of repellents against mosquitoes depends on the vector species as well as the concentration and formulation of the repellent. In this study, we observed the behavioral responses of two night-biting malaria vectors in Thailand, *Anopheles minimus* and *Anopheles dirus*, during daytime and nighttime. We demonstrated that time of observation has a considerable impact on behavioral responses for both species. When optimizing an excito-repellency assay system, time of observation-based testing should be considered in order to prevent an under- or overestimation of behavioral responses.

**Abstract:**

Information on factors influencing the behavioral responses of mosquitoes to repellents is lacking and poorly understood, especially in the *Anopheles* species, night-biting mosquitoes. Our goal was to investigate the impact of different time periods on circadian activity and behavioral responses of two malaria vectors, *Anopheles minimus* and *An. dirus*, to 5% DEET using an excito-repellency test system. Each mosquito species was exposed to the repellent during the daytime (06.00–18.00) and nighttime (18.00–06.00), and time of observation was further divided into four 3-h intervals. Significant escape responses were observed between daytime and nighttime for *An. minimus* in both noncontact and contact tests. *An. dirus* showed statistical differences in contact irritancy escape response, whereas no significant difference was found in noncontact repellency tests. Both mosquito species showed more significantly higher escape responses when exposed to DEET during the afternoon and late in the night. This finding indicates that the time of testing may affect the behavioral responses of mosquitoes to repellents, especially in *An. minimus* and *An. dirus.* A better understanding of nocturnally active mosquito behavioral responses spanning from dusk to dawn would assist in optimizing product development, screening, and effective evaluation.

## 1. Introduction

Malaria is an illness caused by *Plasmodium* parasites. The parasite is transmitted by competent *Anopheles* mosquitoes to humans. It continues to be a serious problem worldwide, including in some provinces in Thailand, especially near international borders with Myanmar and Cambodia, where malaria is endemic [1,2]. These provinces are also the most common destinations for migrant agricultural workers, particularly from Myanmar. These migrant workers are potentially infected and unknowingly transport malaria parasites as they cross the border, resulting in an increased incidence of malaria [3].

In Thailand, there are at least 74 different species of *Anopheles* mosquitoes [4]. Of these, seven species: *Anopheles minimus* Theobald; *An. dirus* (Peyton and Harrison); *An. maculatus* (Theobald); *An. pseudowillmori* (Theobald); *An. aconitus* (Doenitz), *An. baimaii* (Sallum and Peyton); and *An. sawadwongporni* (Rattanarithikul and Green), are regarded as the most important malaria vectors in the country [5,6]. Additionally, various bionomic and host preference studies across the country revealed that these vectors exhibit anthropophilic and exophagic behaviors [4].

Individuals who remain outdoors during evening hours are not under the protection from a bed net or from the indoor residual spraying (IRS), and therefore, they may be readily bitten by malaria-infected *Anopheles* mosquitoes. The use of topical repellents is an alternative option to protect from mosquito bites. One of the most commonly used active ingredient in these topical repellents is *N*,*N* diethyl-*m*-toluamide (DEET). This compound is highly effective and is considered the gold standard for insect repellents [7,8,9,10]. The exact mechanism(s) by which DEET exerts its efficacy against biting flies, however, is unclear. Ditzen et al. (2008) [11] suggested DEET masks the host odor by inhibiting subsets of heterometric odorant receptors in the insect. In addition, DEET has also been shown to block the lactic acid-sensitive olfactory sensory neurons on the antennae of *Aedes aegypti*, thus lowering the attractiveness of a host [12]. Furthermore, DEET has also been shown to inhibit cholinesterase activity, suggesting the compound is not just a behavior-modifying chemical [13] but also has substantial toxic properties against *Ae. aegypti* (L.), *Ae. albopictus* Skuse, and *An. quadrimaculatus* (Say) [14].

The knowledge of physiological and behavioral responses of mosquitoes to insecticides and repellents is crucial for the implementation of vector control strategies and disease transmission prevention programs [9,15,16]. The behavioral responses of mosquitoes to insecticides, natural essential oils, and synthetic repellents have been investigated using excito-repellency test chambers [17,18,19,20,21,22]. Some studies have evaluated the behavioral responses of several species of mosquitoes such as *An. minimus*, *Culex quinquefasciatus*, and *Ae. aegypti* against DEET [23,24]. Choomsang et al. (2018) [25] investigated the influence of daytime periods on the escape response of a laboratory strain *Ae. aegypti* when exposed to DEET, essential oil (hairy basil oil), and deltamethrin. Interestingly, the study found significant escape response of *Aedes* mosquito when exposed to these chemical-based repellents during diurnal periods. Tainchum et al. (2014b) [24] investigated the behavioral responses of field and laboratory *Cx. quinquefasciatus* and *Ae. aegypti* to 5% DEET, wherein significant differences in spatial repellent escape responses between day and nighttime periods were observed for *Cx. quinquefasciatus*, which are nighttime biter mosquitoes, but not for *Ae. aegypti*, which are daytime biter mosquitoes. The effect of repellents on each mosquito’s behavior is still unclear. Moreover, the laboratory efficacy of repellents against mosquitoes depends on mosquito species and the concentration of repellents [26,27] but could also depend on the time when the bioassay was done. Therefore, the time of testing or investigation should be taken into consideration to prevent an underestimate or overestimation of behavioral responses.

Additional information on factors influencing behavioral responses of mosquitoes to repellents is lacking and poorly understood, especially for *Anopheles* species, which are night-biting mosquitoes. To better understand the influence of time on activity and behavior patterns of mosquitoes, the behavioral responses of *An. minimus* and *An. dirus* laboratory strains against DEET were observed during different time periods over a 24-h cycle. In summary, assay conditions, such as time of testing, may significantly influence test outcomes when screening compounds for avoidance action or effects of post-exposure behavior, which might impact vector-borne disease transmission.

## 2. Materials and Methods

### 2.1. Mosquitoes

#### 2.1.1. *Anopheles minimus*

The strain was established in 1993 by the Vector Borne Disease Bureau, Department of Disease Control, Ministry of Public Health, Nonthaburi, Thailand. This colony strain has been maintained in the same laboratory for more than 15 years in the insectary of the Department of Entomology, Faculty of Agriculture, Kasetsart University, Bangkok, Thailand. Routine rearing was done in controlled insectary conditions of 25 ± 5 °C and 80 ± 10% relative humidity, with a 12:12 (L:D) photoperiod following established procedures [20]. Larvae were fed with commercial dried fish food (TetraMin^®^ Tropical Flakes, Germany). Pupae were separated by sex and, following emergence, adult mosquitoes were maintained in a screened cage (30 × 30 × 30 cm) and provided 10% sugar solution on soaked cotton pads for sustenance. For colony maintenance and egg production, self-mating (no artificial insemination is needed) female mosquitoes, aged between 5 and 7 days, were provided human blood via an artificial membrane feeding system [28].

#### 2.1.2. *Anopheles dirus*

*Anopheles dirus* (TMMU strain) was obtained from the Department of Medical Entomology, Faculty of Tropical Medicine, Mahidol University. The strain was collected in Khao Mai Kaeo Subdistrict, Bang Lamung District, Chonburi Province, Eastern Thailand in 1981. Mosquitoes were transferred to the Department of Entomology, Faculty of Agriculture, Kasetsart University, Bangkok, Thailand. *Anopheles dirus* was reared in controlled insectary conditions similar to *An. minimus* as described. Artificial insemination technique was performed for maintaining the colony of *An. dirus* [29].

### 2.2. Repellent-Treated Paper

*N*,*N*-diethyl-*m*-toluamide or *N*,*N*-diethyl-3-methylbenzamide (DEET) was purchased from Sigma-Aldrich Company (St. Louis, MO, USA). Filter paper (Whatman No.1) 14.7 × 17.5 cm was treated with DEET for the excito-repellency assay. Test papers were impregnated with 5% DEET concentration at 182 mg/m^2^ prepared in absolute ethanol. The DEET dose (200 mg/m^2^) was selected as the minimum effective dose to lower the probability that an inadequate amount that may cause a failure of mosquito response [30,31] and eliminate the possibly the DEET might function as an attractant at much lower concentrations [32]. Control papers (without active ingredient) were produced using ethanol only. A precise volume of 2.8 mL of 5% DEET solution was spread evenly on the entire filter paper by a hand-held calibrated serological pipette. Treated papers were air-dried for at least 1 h before use.

### 2.3. Behavioral Tests

An experimental study was designed to compare *An. minimus* and *An. dirus* behavioral response in contact and noncontact chambers exposed to DEET between two periods of time (day and night). Identical test chamber designs were used for all excito-repellency assays [33]. This system was provided to investigate and monitor of mosquito behavioral responses. The excito-repellency apparatus was designed to evaluate behavioral responses of mosquitoes to irritant and/or repellent chemical compounds. The apparatus is divided into two test systems, contact irritancy, and noncontact repellency. The stainless steel outer chamber device measures 34 cm × 32 cm × 32 cm and is connected to an external receiving box for escaping mosquitoes. For the contact chamber, treated papers were attached inside the chamber, which allow direct physical contact, whereas, in the noncontact chamber, screen barriers were installed to prevent mosquito from making direct physical contact with treated papers. The complete design of the excito-repellency assay chambers has been described elsewhere [25,33]. Contact irritancy is defined as the mosquitoes making physical contact with the chemically treated substrate, whereas spatial repellency is defined as the mosquitoes escaping from the test chamber without making physical contact with the chemically treated substrate [19,34].

Three-to-five-day-old, non-blood-fed, nulliparous female *Anopheles* mosquitoes were used in all assays. Mosquitoes were deprived of all nutrition (sugar solution) for 12 h before the start of the assays. Following WHO (2006) [35] procedures, four independent trials (replicates) using 15 female mosquitoes in each chamber with identical controls were conducted for each test condition. Each test trial was composed of four test chambers, two ‘treatment’ designs as either contact or noncontact configurations, each with matching control chambers without active ingredient. Excito-repellency assays were performed during daylight hours between 06.00 and 18.00 separated into 3-h intervals of time (06:00–09:00, 09:00–12:00, 12:00–15:00, and 15:00–18:00) and evening hours between 18:00–06:00 divided into four equal periods (18:00–21:00, 21:00–24:00, 24:00–03:00, and 03:00–06:00). Tests were conducted for 30 min with observations based on the number of mosquitoes escaping from each chamber recorded at one-minute intervals. After each test was completed, the number of knockdown and mortality from each chamber were recorded for mosquitoes that escaped and those that remained inside the chamber. Specimens were separated by response (escape or not) and transferred to clean containers and held to record mortality following 24 h post-test [25].

### 2.4. Data Analysis

Kaplan–Meier survival statistics were used to analyze and interpret the rate of escaped mosquitoes from each chamber [34,36]. Abbott’s formula [37] was used to adjust the number of escaped mosquitoes from the chambers. Choomsang et al. (2018) [25] mentioned that the initial percent escape represents a measure of combined contact excitation (irritancy) and noncontact repellency (termed excito-repellency) in the contact test design. Consequently, the crude contact escape percent was further adjusted to more closely approximate the response resulting from direct tarsal exposure causing irritancy alone to account for any unequal sample sizes between pairings using the equation: (1 − [# contact in test × # noncontact escape/# noncontact in test × # contact escape]) × 100. This calculation, a reciprocal of the Henderson–Tilton (H-T) formula (Henderson and Tilton 1955) [38], was formulated to measure the effects of toxic chemicals on arthropod populations, wherein we compare mosquitoes that successfully escaped the chambers in paired contact and noncontact designs by excluding the repellency effects and provide a better estimate of true contact irritancy [39]. After adjusting, an estimation of percent effect due to contact excitation alone was calculated by dividing the adjusted contact escape (alone) with the pre-adjusted (combined effects) percentage escape for each compound and time interval period. The analysis defined mosquitoes that escaped from the chambers as “dead”, while those did not escape and who remained inside the chambers as “survivors”. A log-rank method [40] was used to compare patterns of escape behavior of two mosquito species, for both contact and noncontact trials, between daytime and nighttime periods using SAS Release 6.10 (SAS Institute, Cary, NC, USA). A statistical significance for all tests was set at 5% (*p* < 0.05).

## 3. Results

We saw significant escape response between daytime and nighttime for both *An. minimus* and *An. dirus* when exposed to DEET-treated chambers in contact experiments (*p* = <0.0001, df = 1, *χ*^2^ = 23.4689 and *p* = 0.0006, df = 1, *χ*^2^ = 11.8982). For noncontact experiments, there was a significant escape response for *An. minimus* (*p* = 0.0233, df = 1, *χ*^2^ = 5.1469), whereas no significant difference was found for *An. dirus* (*p* = 0.2479, df = 1, *χ*^2^ = 1.3351). A greater percentage of escaped *An. dirus* was observed during daytime from noncontact (11.29–84.21%) compared to contact trials (6.56–66.67 (Table 1), although the difference is not significant (*p* = 0.2479, df = 1, *χ*^2^ = 1.3351) (Table S1).

Corrected percentage of *An. minimus* escape response to DEET after using Abbott’s formula was observed in both daytime and nighttime experiments from both the noncontact trial (32.92–50.98% and 49.70–55.29%, respectively) and contact trial (20.99–34.00% and 53.34–70.77%, respectively). The highest corrected escape response was observed at 18.00–21.00 (50.98%) in the noncontact trial and at 21.00–24.00 (70.77%) in the contact trial, whereas the lowest corrected escape response was observed at 15.00–18.00 (32.92%) in the noncontact trial and at 12.00–15.00 (20.99%) in the contact trial (Table 2). For *An. dirus*, DEET produced a higher corrected percentage of escape response in both daytime and nighttime periods from the noncontact trial (56.61–72.27% and 9.99–69.23%, respectively) compared with the contact trial (33.78–42.59% and 35.19–37.93%, respectively). The highest corrected escape response was observed at 06.00–09.00 (72.27%) in the noncontact trial and at 15.00–18.00 (42.59%) in the contact trial, whereas the lowest corrected escape response was observed at 24.00–03.00 (9.99%) in the noncontact trial and at 06.00–09.00 (33.78%) in the contact trial. Moreover, the corrected percentage escaped mosquitoes in the noncontact treatment test at 03.00–06.00 and in contact treatment tests at 24.00–03.00 and 03.00–06.00 were not seen (not applicable) (Table 3).

There were no significant differences in escape seen between four different time periods for *An. minimus* (noncontact-daytime: *p* = 0.2504–0.8850, df = 1, *χ*^2^ = 0.0209–1.3213; contact-daytime: *p* = 0.0698–0.7113, df = 1, *χ*^2^ = 0.1370–3.2866; noncontact-nighttime: *p* = 0.4541–0.9821, df = 1, *χ*^2^ = 0.0005–0.5603; contact-nighttime: *p* = 0.0835–0.8484, df = 1, *χ*^2^ = 0.0365–2.9961). However, for *An. dirus*, there was a significant difference in escape seen between the 06.00–09.00 and 15.00–18.00 periods (*p* = 0.0046, df = 1, *χ*^2^ = 8.0413) and a similar difference was found between the 06.00–09.00 and 09.00–12.00 periods (*p* = 0.0278, df = 1, *χ*^2^ = 4.8387) in the noncontact treatment trial (Table 4 and Table 5).

For *An. minimus*, there were statistically significant differences between treatment and control trials in both noncontact and contact trials in daytime hours, and similar results were found in nighttime testing. A significant difference was found between noncontact and contact trials during 12.00–15.00 (Table S2). DEET produced similarly marked escape responses for *An. dirus*. There were statistically significant differences between treatment and control trials in both noncontact and contact trials in daytime hours and similar results were found in nighttime testing, except the 24.00–0300 (*p* = 0.8416, df = 1, *χ*^2^ = 0.0400) and 03.00–06.00 (*p* = 0.3137, df = 1, *χ*^2^ = 1.0151) periods of noncontact trials. No significant differences were found between noncontact treatment and contact treatment trials during 15.00–18.00 and 03.00–06.00 periods (Table S3).

Overall, a greater percentage of knockdown and mortality was observed in mosquitoes that failed to escape inside the treatment chamber compared to the untreated control. For *An. minimus*-day, no 30 min knockdown and very low mortality (0–3.22%) was recorded in both escape and remain groups of mosquitoes in both the noncontact and contact control chambers (Tables S4 and S5). The contact test produced the greatest knockdown (73.01%) during daytime periods. Over all test period intervals, knockdown responses for mosquitoes that did not escape in both the noncontact and contact treatment tests during daytime periods ranged from 35.00–45.90% and 56.90–73.01%, respectively, and during nighttime periods from 0–25.00% and 0–27.59%, respectively. For *An. dirus*, a few cases of 30 min knockdown(s) (1.63%) or 24-h mortality (1.63%) were recorded in noncontact control trials and only 1.67% mortality was observed from mosquitoes that did not escape during nighttime testing (Tables S4–S5). Knockdown responses for mosquitoes that did not escape from daytime testing in both noncontact and contact tests ranged from 1.67–38.98% and 22.58–42.86%, respectively, and during nighttime periods from 4.84–23.33% and 28.81–55.17%, respectively (Table 2 and Table 3).

**Figure 1:**
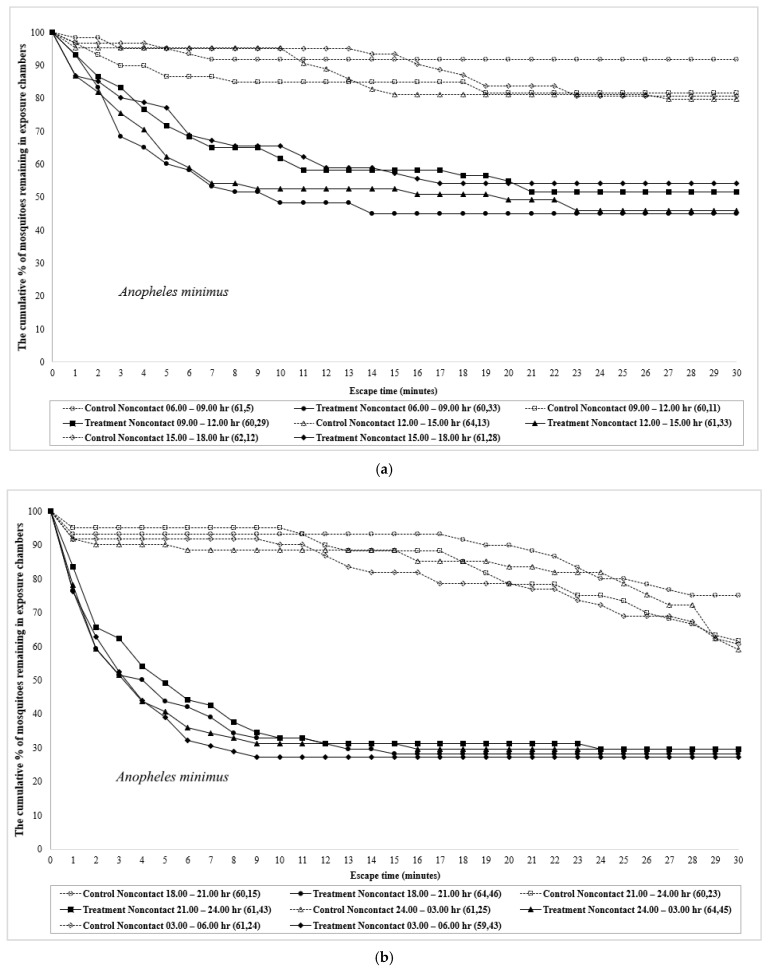

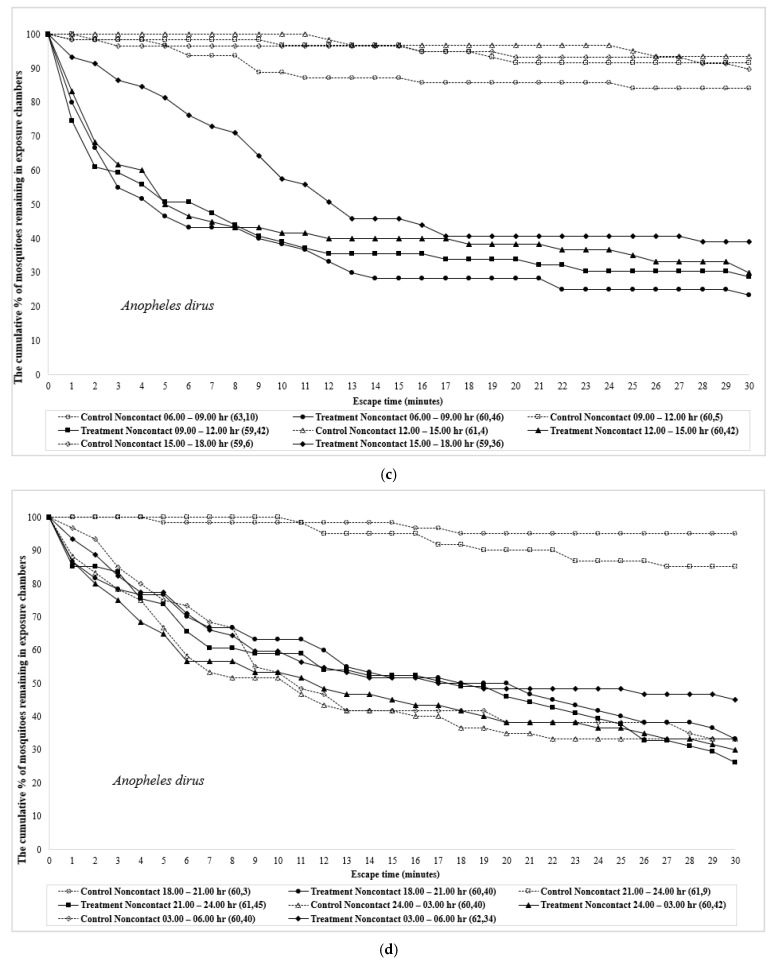
Kaplan–Meier survival curves showing escape responses by (**a**) *Anopheles minimus*-day; (**b**) *Anopheles minimus*-night; (**c**) *Anopheles dirus*-day; and (**d**) *Anopheles dirus*-night between different time intervals in noncontact trials when exposed to DEET. The number of individuals subjected to test (first number) and those escaped (second number) are given in parentheses.

**Figure 2:**
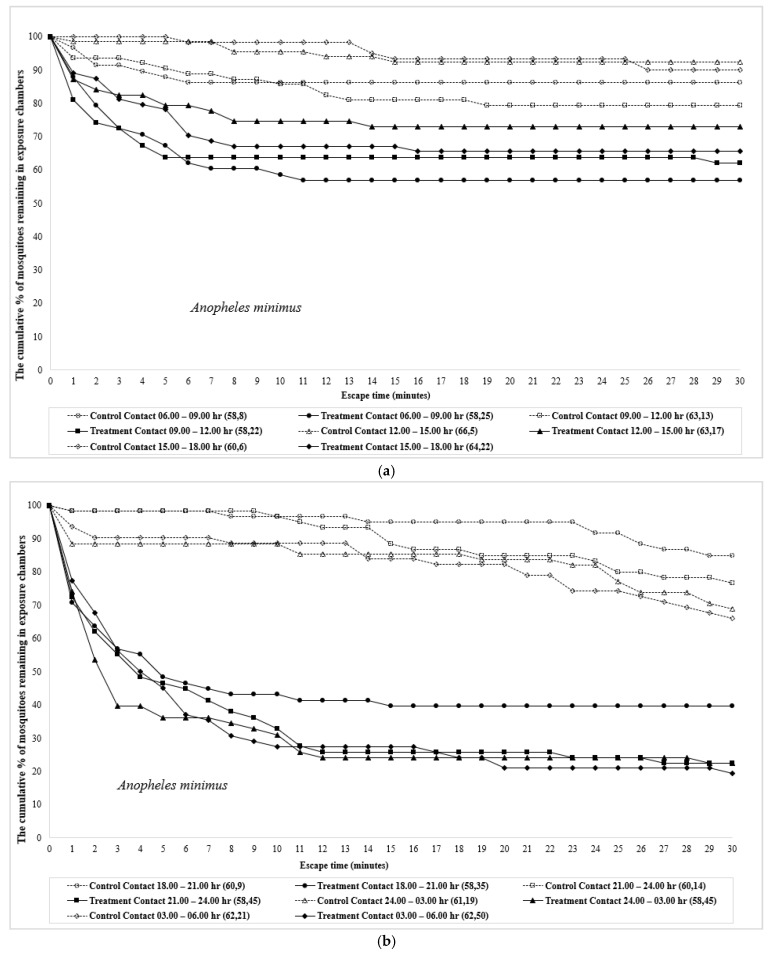

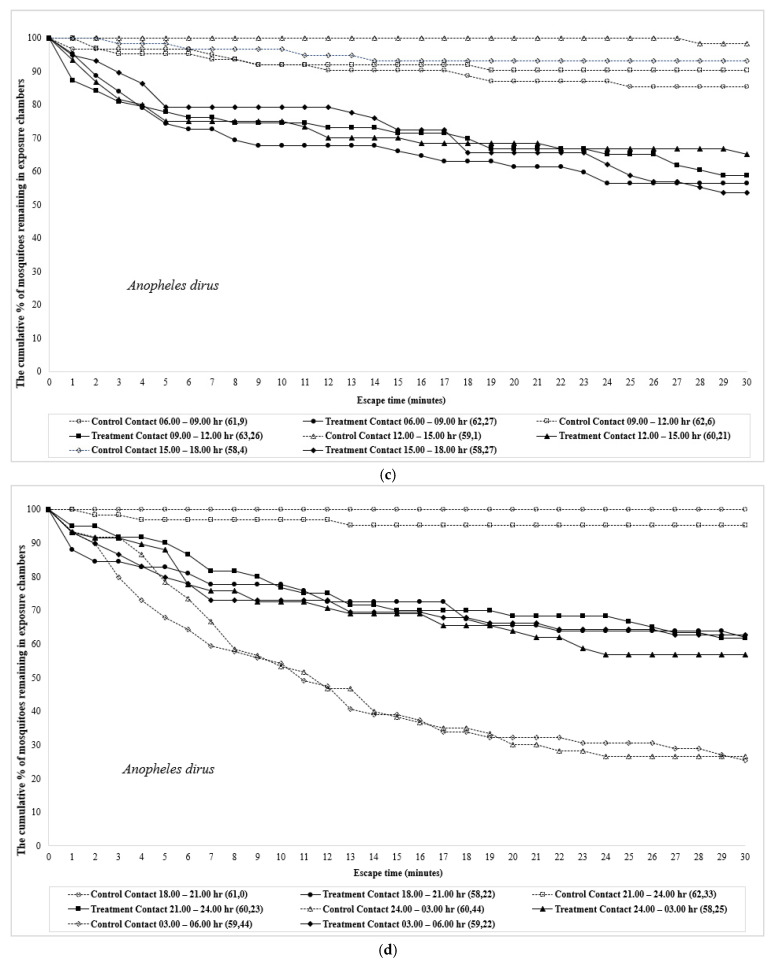
Kaplan–Meier survival curves showing escape responses by (**a**) *Anopheles minimus*-day; (**b**) *Anopheles minimus*-night; (**c**) *Anopheles dirus*-day; and (**d**) *Anopheles dirus*-night between different time intervals in contact trials when exposed to DEET. The number of individuals subjected to test (first number) and those escaped (second number) are given in parentheses.

Patterns of mosquitoes that did not escape and remained inside the test chambers in contact and noncontact tests of *An. minimus* and *An. dirus* are presented in Figure 1 and Figure 2. The *y*-axis represents the cumulative percentage of mosquitoes remaining inside the exposure chamber after each one-minute interval. The area above the curve represents the total cumulative percentage of escaping mosquitoes. The data used in Figure 1 and Figure 2 were not adjusted to exclude the estimated repellency effect; thus, the escape percentage represents a combination of contact irritancy and spatial repellency. DEET showed stronger repellency and irritancy actions during nighttime compared with daytime hours in *An. minimus*, whereas the greatest repellency was demonstrated in both daytime and nighttime periods in *An. dirus*. Interestingly, a greater repellency was observed in control than treatment trials in 24.00–03.00 and 03.00–06.00 periods (Tables S2 and S3).

## 4. Discussion

Excito-repellency test chambers were used to evaluate escape response behavior (behavioral avoidance) of *An. minimus* and *An. dirus* during daytime and nighttime periods when exposed to 5% DEET, a synthetic active ingredient used in a wide range of commercial insect repellent products. Our study aimed to find if time of testing can influence the behavioral responses of mosquitoes, which might be critical for the proper interpretation of excito-repellency data. Our findings indicated clear differences in avoidance response for both species depending on the time of testing with stronger escape activity occurring during the nighttime hours. The greater impact of time was seen in spatial repellency than contact irritancy due to “true irritancy effect” (percentage of escape present in contact excitation (excluding the repellency effect)). Normally, by contact test design, contact irritancy actually represents a measure of combined contact irritancy and noncontact repellency [39].

Our findings found differences in the mosquito escape response between daytime and nighttime test periods in noncontact trial only for *An. minimus*, whereas no significant differences were observed for *An. dirus*. However, significant escape responses between day and night periods were observed in contact trials for both *An. minimus* (*p* = 0.0001) and *An. dirus* (*p* = 0.0006) species. A higher corrected percentage of *An. minimus* escape because of DEET was observed in the nighttime experiment (49.93%) from the noncontact trial compared with the daytime experiment (43.56%). Conversely, DEET produced a higher corrected percentage of *An. dirus* escape in the daytime period (82.20%) from the noncontact trial compared with the nighttime period (53.60%), but a higher percentage of escape was found in nighttime periods compared with daytime periods from the noncontact control. It was assumed that more mosquitoes would be repelled during nighttime, when these species are more active, but our result showed that this was not always the case. Thus, careful consideration must be taken when interpreting contact or noncontact experiment results for different species of mosquito. Greater escape activities from untreated controls during the nighttime tests were demonstrated. These findings correspond to a previous study by Tainchum et al. (2014b) [24], who showed that both in field and laboratory experiments, *Cx. quinquefasciatus*, a night-biter mosquito, had a higher percentage of escape in nighttime periods compared to daytime periods. In this study, with all other environment and experimental factors, such as age of mosquitoes, physiology, ambient temperature, relative humidity, and illumination, being equal, exposure to DEET clearly influenced behavior response and affected normal innate (circadian) activity in the two species of night mosquitoes tested (*An. minimus* and *An. dirus* laboratory strains). The impact of time could be seen in irritancy and/or repellency responses.

There were no significant differences in escape seen between two morning intervals (06.00–09.00 and 09.00–12.00) or between the two afternoon test periods (12.00–15.00 and 15.00–18.00). Moreover, there were no significant differences in escape seen between two early-night intervals (18.00–21.00 and 21.00–24.00) or between the two late-night test periods (24.00–03.00 and 03.00–06.00) from our study. This may be caused by various different factors, such as light intensity, temperature, relative humidity, and the human host cues from the operator of the experiment [25].

Many factors are involved in malaria transmission, such as mosquito density and habitat associations, vector competence, dusk to dawn activity patterns, and host-seeking/feeding habits [41]. *Anopheles* mosquitoes are typically evening blood feeders, active during various periods between dusk and dawn [42]. Under natural conditions, *An. minimus* is an important malaria vector in Thailand and can display both zoophilic and anthropophilic/phagic behaviors [6]. The biting patterns of *An. minimus* in western Thailand have shown peak indoor activity patterns in the early morning hours (01.00–02.00) [43]. Our investigations with DEET exposure found a higher percentage of a long-colonized *An. minimus* escaping between 24.00–06.00 in both control and treatment trials, which corresponds to the typical trophic behavior in the field. For *An. dirus*, an important and highly competent malaria vector in Thailand [6,44], this species is primarily anthropophilic but mainly exophagic, which complicates controlling transmission using insecticides [45,46,47]. Whereas Tananchai et al. (2012) [48] demonstrated exophagic and zoophilic behaviors of *An. dirus* with highest biting peak between 19.00 and 20.00 and a smaller peak between 02.00 and 03.00, our study showed the highest percentage of escape responses in the 24.00–03.00 and 03.00–06.00 periods in both contact and noncontact control trials. A possible explanation could be the existence of some variability in the laboratory strain of mosquitoes. Both mosquitoes used in this study have been colonized in laboratory settings for many years. Nevertheless, the artificial day–night setting, which is imitated by the use of artificial light, during the rearing cycle would have preserved the internal biological clock and their 24-h activity.

In our study, a greater percentage of escaped *An. dirus* mosquitoes was found during 24.00–03.00 and 03.00–06.00 in noncontact trials, whereas in contact trials, the escape responses were lower. As expected, a greater percentage of knockdown and mortality were observed in contact treatment chambers, and the level of knockdown and mortality were higher in mosquitoes that did not display an escape response, as they were exposed to DEET much longer [25].

In this study, strong repellency was found during 12.00–15.00 (afternoon time) for *An. minimus*, whereas three periods (06.00–09.00, 12.00–15.00, and 15.00–18.00) of daytime tests and three periods of night tests (18.00–21.00, 21.00–24.00, and 24.00–03.00) showed statistical differences between noncontact and contact tests. The causes of behavioral responses for these observations are still unclear (Tables S2 and S3). Possibly, differences of mosquito species/strains or physiology such as odorant factors might be the cause of the different behavioral responses during daytime and nighttime. DNA microarray analysis of female *An. gambiae* revealed that different light conditions affected the expression of olfaction genes, especially at the transition from the light:dark cycle to completely dark periods [49]. In further studies, Rund et al. (2012) [50] reported that the effects of circadian rhythms on flight activity of *An. gambiae* were strain- and sex-specific.

## 5. Conclusions

This study indicates that time of testing may influence the result of mosquito behavioral assay, especially for *An. minimus* and *An. dirus*, as demonstrated here. The different time of testing between day and night might cause different behavioral responses, as with *An. minimus*, or effects, as with *An. dirus*. A better understanding of nocturnally active mosquito behavioral responses spanning from dusk to dawn would assist in optimizing product screening, development testing, and effective evaluation. As shown in previous studies [24,28], mosquitoes’ behavioral responses showed both repellency and irritancy (combined effect of contact irritancy and repellency) actions when exposed to DEET, and different results were found at different testing times. Therefore, consideration for the time of testing in experimental designs should be made. This would depend on the test species of interest and possibly whether colonized strains or wild-caught populations are used. This study used two long-term colonized strains of anophelines; therefore, care must be taken when extrapolating controlled laboratory mosquitoes with response in natural populations.

## Figures and Tables

**Table 1:** The average percentage of escaped, knockdown, and 24-h mortality of *Anopheles minimus* and *Anopheles dirus* from DEET exposure chambers conducted between day and night using the excito-repellency assay system.

Time	Species	Test Design	No. Mosquitoes		Percent Escape (Mean % Escape ± SE) [^a^]		Corrected % Escape ^b^	% Knockdown		% Mortality	
			DEET	Control	DEET	Control		E	R	E	R
Day	*Anopheles minimus*	Noncontact	61	62	52.46 (13.12 ± 0.24)	16.13	43.56	6.56	39.34	6.56	13.11
		Contact	62	64	22.58 (5.65 ± 0.26) [0] ^a^	15.63	8.24	9.68	77.42	1.61	53.22
	*Anopheles dirus*	Noncontact	57	62	84.21 (21.05 ± 0.78)	11.29	82.20	3.51	15.79	7.02	1.75
		Contact	60	61	28.33 (7.08 ± 0.57) [0] ^a^	6.56	23.30	5.00	38.33	1.67	45.00
Night	*Anopheles minimus*	Noncontact	68	60	69.12 (17.28 ± 0.37)	38.33	49.93	5.88	14.70	1.47	7.35
		Contact	57	60	66.67 (16.67 ± 0.48) [0] ^a^	26.67	54.55	0	10.53	0	8.77
	*Anopheles dirus*	Noncontact	63	61	63.49 (15.87 ± 0.33)	21.31	53.60	7.93	11.11	1.59	1.59
		Contact	58	59	39.66 (9.92 ± 0.28) [0] ^a^	35.59	6.32	10.34	44.82	8.62	25.86

E = escaped from the exposure chamber, R = remained inside the exposure chamber, ^a^ Final estimated percentage of escaped mosquitoes from the contact chamber adjusted using paired noncontact escape to exclude repellency effect (see Materials and Methods for details). ^b^ Percent escape response (at 30 min) to DEET treatment adjusted using the Abbott’s formula. SE = Standard error, calculated using Excel (Microsoft Office 2016).

**Table 2:** The average percent escape, percent knockdown, and 24-h mortality of *Anopheles minimus* exposed to 5% DEET at different time periods of day and night using the excito-repellency assay system.

Time	Test Design	Time Period	No. Mosquitoes		Percent Escape (Mean % Escape ± SE) [^a^]		Corrected % Escape ^b^	% Knockdown		% Mortality	
			DEET	Control	DEET	Control		E	R	E	R
Day	Noncontact	06.00–09.00	60	61	55.00 (13.75 ± 0.27)	8.20	50.98	11.67	41.67	1.67	6.67
		09.00–12.00	60	60	48.33 (12.08 ± 0.51)	18.33	36.73	3.33	35.00	6.67	8.33
		12.00–15.00	61	64	54.10 (13.53 ± 0.20)	20.31	42.40	11.47	40.98	0	16.39
		15.00–18.00	61	62	45.90 (11.48 ± 0.45)	19.35	32.92	11.47	45.90	0	14.75
	Contact	06.00–09.00	58	58	43.10 (10.78 ± 0.39) [0] ^a^	13.79	34.00	13.79	56.90	6.89	50.00
		09.00–12.00	58	63	37.93 (9.48 ± 0.57) [0] ^a^	20.63	21.80	27.58	62.07	22.41	39.65
		12.00–15.00	63	66	26.98 (6.75 ± 0.25) [0] ^a^	7.58	20.99	22.22	73.01	3.17	73.01
		15.00–18.00	64	60	34.38 (8.60 ± 0.52) [0] ^a^	10.00	27.09	21.87	65.62	18.75	53.13
Night	Noncontact	18:00–21:00	64	60	71.88 (17.97 ± 0.41)	25.00	62.51	5.68	25	0	18.75
		21:00–24:00	61	60	70.49 (17.62 ± 0.36)	38.33	52.15	0	0	0	9.83
		24:00–03:00	64	61	70.31 (17.58 ± 0.70)	40.98	49.70	7.81	17.19	3.12	17.18
		03:00–06:00	59	61	72.88 (18.22 ± 0.87)	39.34	55.29	3.39	23.73	0	8.47
	Contact	18:00–21:00	58	60	60.34 (15.09 ± 0.47) [0] ^a^	15.00	53.34	5.17	27.59	1.72	18.95
		21:00–24:00	58	60	77.59 (19.40 ± 0.34) [7.09] ^a^	23.33	70.77	0	0	0	6.77
		24:00–03:00	58	61	77.59 (19.40 ± 0.60) [7.27] ^a^	31.15	67.45	6.90	17.24	6.89	10.34
		03:00–06:00	62	62	80.65 (20.16 ± 0.52) [7.76] ^a^	33.87	70.74	1.61	16.13	0	11.29

E = escaped from the exposure chamber, R = remained inside the exposure chamber, ^a^ Final estimated percentage of escaped mosquitoes from the contact chamber adjusted using paired noncontact escape to exclude repellency effect (see Materials and Methods for details). ^b^ Percent escape response (at 30 min) to DEET treatment adjusted using the Abbott’s formula. SE = Standard error, calculated using Excel (Microsoft Office 2016).

**Table 3:** The average percent escape, percent knockdown, and 24-h mortality of *Anopheles dirus* exposed to 5% DEET at different time periods of day and night using the excito-repellency assay system.

Time	Test Design	Time Period	No. Mosquitoes		Percent Escape (Mean % Escape ± SE) [^a^]		Corrected % Escape ^b^	% Knockdown		% Mortality	
			DEET	Control	DEET	Control		E	R	E	R
Day	Noncontact	06.00–09.00	60	63	76.67 (19.17 ± 0.37)	15.87	72.27	0	1.67	6.67	0
		09.00–12.00	59	60	71.19 (17.80 ± 0.38)	8.33	68.57	0	1.67	0	1.67
		12.00–15.00	60	61	70.00 (17.50 ± 0.57)	6.56	67.89	0	5.00	0	1.67
		15.00–18.00	59	59	61.02 (15.26 ± 0.36)	10.17	56.61	3.39	38.98	0	3.39
	Contact	06.00–09.00	62	61	43.55 (10.89 ± 0.68) [0] ^a^	14.75	33.78	6.45	22.58	0	24.19
		09.00–12.00	63	62	41.27 (10.32 ± 0.84) [0] ^a^	9.68	34.98	7.93	42.86	1.58	26.98
		12.00–15.00	60	59	35.00 (8.75 ± 0.32) [0] ^a^	1.69	33.88	0	30.00	0	8.33
		15.00–18.00	58	58	46.55 (11.64 ± 0.72) [0] ^a^	6.90	42.59	13.79	41.38	0	22.41
Night	Noncontact	18:00–21:00	60	60	66.67 (16.67 ± 0.63)	5.00	64.92	11.67	11.67	1.67	11.67
		21:00–24:00	61	61	73.77 (18.44 ± 0.63)	14.75	69.23	0	9.83	0	1.64
		24:00–03:00	60	60	70.00 (17.50 ± 0.51)	66.67	9.99	10.00	23.33	3.33	11.67
		03:00–06:00	62	60	54.84 (13.71 ± 0.70)	66.67	N/A	11.29	4.84	0	0
	Contact	18:00–21:00	58	61	37.93 (9.48 ± 0.54) [0] ^a^	0	37.93	12.07	48.26	3.45	10.34
		21:00–24:00	60	62	38.33 (9.58 ± 0.27) [0] ^a^	4.84	35.19	15.00	40.00	8.33	31.67
		24:00–03:00	58	60	43.10 (10.78 ± 0.47) [0] ^a^	73.33	N/A	12.07	55.17	15.52	31.03
		03:00–06:00	59	59	37.29 (9.32 ± 0.64) [0] ^a^	74.58	N/A	6.78	28.81	1.69	6.78

E = escaped from the exposure chamber, R = remained inside the exposure chamber, N/A = not applicable (escaped mosquitoes from control chambers more than those that escaped the treated chambers). ^a^ Final estimated percentage of escaped mosquitoes from the contact chamber adjusted using paired noncontact escape to exclude repellency effect (see Materials and Methods for details). ^b^ Percent escape response (at 30 min) to DEET treatment adjusted using the Abbott’s formula. SE = Standard error, calculated using Excel (Microsoft Office 2016).

**Table 4:** Log-rank tests comparing diel quarterly time periods in daytime within species and treatments for *Anopheles minimus* and *Anopheles dirus*.

Mosq. Species	Test Conditions	*p* Value of Time Period Comparisons (Chi-Square Value)					
		D1 vs. D2	D1 vs. D3	D1 vs. N4	D2 vs. D3	D2 vs. D4	D3 vs. D4
*Anopheles minimus*	noncontact control	0.1018 (2.6778)	0.0662 (3.3747)	0.0888 (2.8966)	0.8507 (0.0354)	0.9746 (0.0010)	0.8015 (0.0632)
	noncontact treatment	0.3317 (0.9421)	0.8850 (0.0209)	0.2504 (1.3213)	0.4253 (0.6356)	0.8296 (0.0463)	0.3168 (1.0019)
	contact control	0.3620 (0.8310)	0.2360 (1.4045)	0.4552 (0.5577)	0.0307* (4.6702)	0.0815 (3.0353)	0.6638 (0.1890)
	contact treatment	0.7113 (0.1370)	0.0698 (3.2866)	0.2873 (1.1320)	0.1777 (1.8164)	0.5369 (0.3813)	0.4033 (0.6984)
*Anopheles dirus*	noncontact control	0.1907 (1.7121)	0.0894 (2.8846)	<0.0001 * (28.3614)	0.6939 (0.1549)	<0.0001 * (48.0177)	<0.0001 * (61.6943)
	noncontact treatment	0.6513 (0.2043)	0.3907 (0.7368)	0.0046 * (8.0413)	0.7344 (0.1151)	0.0278 * (4.8387)	0.0519 (3.7785)
	contact control	0.4073 (0.1084)	0.0093 * (4.8862)	0.1771 (0.7715)	0.0587 (3.5724)	0.5717 (0.3198)	0.1612 (1.9626)
	contact treatment	0.7834 (0.0756)	0.4031 (0.6990)	0.9979 (0.0000)	0.5305 (0.3935)	0.6979 (0.1510)	0.3618 (0.8318)

Periods (hours): D1 = 06.00–09.00, D2 = 09.00–12.00, D3 = 12.00–15.00, D4 = 15.00–18.00. * Statistical significance set at *p* < 0.05.

**Table 5:** Log-rank tests comparing diel quarterly time periods in nighttime within species and treatments for *Anopheles minimus* and *Anopheles dirus*.

Mosq. Species	Test Conditions	*p* Value of Time Period Comparisons (Chi-Square Value)					
		N1 vs. N2	N1 vs. N3	N1 vs. N4	N2 vs. N3	N2 vs. N4	N3 vs. N4
*Anopheles minimus*	noncontact control	0.1258 (2.3436)	0.0946 (2.7945)	0.0924 (2.8317)	0.8914 (0.0186)	0.8515 (0.0350)	0.9809 (0.0006)
	noncontact treatment	0.6623 (0.1907)	0.9821 (0.0005)	0.7903 (0.0707)	0.6444 (0.2131)	0.4541 (0.5603)	0.8174 (0.0533)
	contact control	0.2312 (1.4337)	0.0320 * (4.5962)	0.0128 * (6.1964)	0.3364 (0.9239)	0.1919 (1.7029)	0. 7393 (0.1108)
	contact treatment	0.1346 (2.2382)	0.0835 (2.9961)	0.0855 (2.9568)	0.7596 (0.0937)	0.7730 (0.0832)	0.8484 (0.0365)
*Anopheles dirus*	noncontact control	0.0793 (3.9578)	<0.0001 * (102.4690)	<0.0001 * (101.6812)	<0.0001 * (41.3270)	<0.0001 * (40.0028)	0.6367 (0.2215)
	noncontact treatment	0.4934 (0.4691)	0.4759 (0.5083)	0.3346 (0.9311)	0.9248 (0.0089)	0.1002 (2.7031)	0.0986 (0.7285)
	contact control	0.0832 (3.0004)	<0.0001 * (74.5956)	<0.0001 * (76.9401)	<0.0001 * (62.2740)	<0.0001 * (64.9718)	0.7145 (0.1338)
	contact treatment	0.2099 (0.0051)	0.0672 (0.2218)	0.2139 (0.0019)	0.5440 (0.3682)	0.9291 (0.0079)	0.6470 (0.2097)

Periods (hours): N1 = 18.00–21.00, N2 = 21.00–24.00, N3 = 24.00–03.00, N4 = 03.00–06.00. * Statistical significance set at *p* < 0.05.

## Supplementary Materials

The following are available online at https://www.mdpi.com/article/10.3390/insects12100867/s1, Table S1: Log-rank tests analysis comparing pattern of escaped mosquitoes in each population between day and night trials, Table S2: Log-rank tests comparing pattern of *Anopheles minimus* escape responses in noncontact control, noncontact treatment, contact control and contact treatment by time interval, Table S3: Log-rank tests comparing pattern of *Anopheles dirus* escape responses in noncontact control, noncontact treatment, contact control and contact treatment by time interval, Table S4: The average percent escape, percent knockdown and 24-hr mortality of *Anopheles minimus* and *Anopheles dirus* from untreated control chambers conducted between day and night periods using the excito-repellency assay system, Table S5: The average percent escape, percent knockdown and 24-hr mortality of *Anopheles minimus* and *Anopheles dirus* from untreated controls at different time periods of day and night..
