# Supplementary material for: Time of Test Periods Influence the Behavioral Responses of Anopheles minimus and Anopheles dirus (Diptera: Culicidae) to DEET
Source: Insects. 2021 Sep 24;12(10):867. doi: 10.3390/insects12100867 (PMC8540783; doi:10.3390/insects12100867)
Supplement: Supplementary file 1 [file insects-12-00867-s001.zip › insects-1344764-supplementary.pdf]

Table S1. Log-rank tests analysis comparing pattern of escaped mosquitoes in each population between day and night trials.

**Populations**	***P* value of day and night period comparisons (Chi-square value)**			
	**Noncontact**		**Contact**	
	**Treatment**	**Control**	**Treatment**	**Control**
Anopheles minimus	0.0233* (5.1469)	0.0083* (6.9734)	<0.0001* (23.4689)	0.1577 (1.9959)
Anopheles dirus	0.2479 (1.3351)	0.0001* (2.4799)	0.0006* (11.8982)	0.0001* (14.7728)#

* Statistical significance set at $P < 0.05$.

Table S2. Log-rank tests comparing pattern of *Anopheles minimus* escape responses in noncontact control, noncontact treatment, contact control and contact treatment by time interval.

**Period**	***P* value of time period comparisons (Chi-square value)**			
	**NC vs NT**	**NC vs CC**	**NT vs CT**	**CC vs CT**
06.00–09.00	<0.0001* (31.0538)	0.3154 (1.0081)	0.3007 (1.0710)	0.0006* (11.9327)
09.00–12.00	0.0007* (11.5841)	0.7716 (0.0842)	0.4947 (0.4664)	0.0227* (5.1885)
12.00–15.00	<0.0001* (17.7947)	0.0389* (4.2632)	0.0036* (8.4597)	0.0026* (9.0606)
15.00–18.00	0.0005* (12.0029)	0.1445 (2.1292)	0.2504 (1.3212)	0.0006* (11.7164)
18.00–21.00	<0.0001* (35.1767)	0.1526 (2.0463)	0.2971 (1.0873)	<0.0001* (31.3814)
21.00–24.00	<0.0001* (22.6671)	0.0918 (2.8423)	0.4268 (0.6316)	<0.0001* (49.3873)
24.00–03.00	<0.0001* (19.1351)	0.3418 (0.9038)	0.4173 (0.6579)	<0.0001* (33.4213)
03.00–06.00	<0.0001* (24.0661)	0.5532 (0.3516)	0.7823 (0.0764)	<0.0001* (38.9952)

NC = noncontact control trial, NT = noncontact treatment trial,
CC = contact control trial, CT = contact treatment trial.
* Statistical significance set at $P < 0.05$.

Table S3. Log-rank tests comparing pattern of *Anopheles dirus* escape responses in noncontact control, noncontact treatment, contact control and contact treatment by time interval.

**Test condition**	***P* value of time period comparisons (Chi-square value)**			
**Period**	**NC vs NT**	**NC vs CC**	**NT vs CT**	**CC vs CT**
06.00–09.00	<0.0001* (53.1583)	0.8508 (0.4755)	<0.0001* (16.8322)	0.0003* (16.2538)
09.00–12.00	<0.0001* (55.1260)	0.7676 (0.0873)	0.0003* (13.3944)	<0.0001* (16.2367)
12.00–15.00	<0.0001* (57.6818)	0.1796 (1.8011)	0.0001* (14.9282)	<0.0001* (22.2121)
15.00–18.00	<0.0001* (49.6023)	0.1971 (1.6638)	0.0850 (2.9658)	<0.0001* (22.7456)
18.00–21.00	<0.0001* (101.1283)	0.0782 (1.6340)	<0.0001* (8.3331)	<0.0001* (28.5888)
21.00–24.00	<0.0001* (47.5364)	0.0735 (3.2021)	0.0001* (14.7947)	<0.0001* (20.0390)
24.00–03.00	0.8416 (0.0400)	0.9575 (0.0028)	0.0019* (9.6027)	0.0011* (10.7236)
03.00–06.00	0.3137 (1.0151)	0.3730 (0.7937)	0.0657 (3.3873)	0.0001* (14.5832)

NC = noncontact control trial, NT = noncontact treatment trial,
CC = contact control trial, CT = contact treatment trial.
* Statistical significance set at $P < 0.05$.

Table S4. The average percent escape, percent knockdown and 24-hr mortality of *Anopheles minimus* and *Anopheles dirus* from untreated control chambers conducted between day and night periods using the excito-repellency assay system.

**Time**	**Species**	**Test design**	**No. mosquitoes**	**Percent escape (mean % escape±SE)**	**% Knockdown**		**% Mortality**	
					**E**	**R**	**E**	**R**
Day	*Anopheles minimus*	NC	62	16.13 (4.03±0.26)	0	0	0	1.61
		CC	64	15.63 (3.91±0.20)	0	0	1.56	0
	Anopheles dirus	NC	62	11.29 (2.82±0.26)	0	0	0	0
		CC	61	6.56 (1.64±0.24)	0	0	0	0
Night	*Anopheles minimus*	NC	60	38.33 (9.58±0.95)	0	0	0	0
		CC	60	26.67 (6.67±0.88)	0	0	0	0
	Anopheles dirus	NC	61	21.31 (5.33±0.81)	0	0	0	0
		CC	59	35.59 (8.90±1.23)	0	0	1.69	0

E = escaped from the exposure chamber, R = remained inside the exposure chamber

SE = Standard error, calculated using excel (Microsoft office 2016)

Table S5. The average percent escape, percent knockdown and 24-hr mortality of *Anopheles minimus* and *Anopheles dirus* from untreated controls at different time periods of day and night.

**Time**	**Test design**	**Time period**	**No. mosq.**	**Percent escape (mean % escape±SE)**	**% Knockdown**		**% Mortality**		**No. mosq.**	**Percent escape (mean % escape±SE)**	**% Knockdown**		**% Mortality**	
					**E**	**R**	**E**	**R**			**E**	**R**	**E**	**R**
				Anopheles minimus						*Anopheles dirus*				
Day	Noncontact	06.00–09.00	61	8.20 (2.05±0.10)	0	0	0	0	63	15.87 (3.97±0.12)	0	0	0	0
		09.00–12.00	60	18.33 (4.58±0.32)	0	0	0	1.67	60	8.33 (2.08±0.21)	0	0	0	0
		12.00–15.00	64	20.31 (5.08±0.19)	0	0	1.56	3.12	61	6.56 (1.64±0.24)	0	0	0	0
		15.00–18.00	62	19.35 (4.84±0.17)	0	0	0	3.22	59	10.17 (2.54±0.13)	0	0	0	0
	Contact	06.00–09.00	58	13.79 (3.45±0.18)	0	0	0	0	61	14.75 (3.69±0.26)	0	0	0	0
		09.00–12.00	63	20.63 (5.16±0.30)	0	0	1.58	0	62	9.68 (2.42±0.12)	0	0	0	0
		12.00–15.00	66	7.58 (1.90±0.18)	0	0	1.51	3.03	59	1.69 (0.42±0.11)	0	0	0	0
		15.00–18.00	60	10.00 (2.50±0.41)	0	0	0	1.67	58	6.90 (1.73±0.32)	0	0	0	0
Night	Noncontact	18:00–21:00	60	25.00 (6.25±0.59)	0	0	0	0	60	5.00 (1.25±0.11)	0	0	0	0
		21:00–24:00	60	38.33 (9.58±0.71)	0	0	0	0	61	14.75 (3.69±0.40)	0	1.63	0	1.63
		24:00–03:00	61	40.98 (10.25±0.67)	0	0	0	0	60	66.67 (16.67±1.17)	0	0	0	0
		03:00–06:00	61	39.34 (9.84±0.66)	0	0	0	0	60	66.67 (16.67±0.18)	0	0	0	0
	Contact	18:00–21:00	60	15.00 (3.75±0.44)	0	0	0	0	61	0 (0)	0	0	0	0
		21:00–24:00	60	23.33 (5.83±0.51)	0	0	0	1.67	62	4.84 (1.21±0.10)	0	0	0	0
		24:00–03:00	61	31.15 (7.79±0.69)	0	0	0	0	60	73.33 (18.33±0.58)	0	0	0	1.67
		03:00–06:00	62	33.87 (8.47±0.51)	0	0	0	0	59	74.58 (18.65±0.18)	0	0	0	0

E = escaped from the exposure chamber, R = remained inside the exposure chamber

SE = Standard error, calculated using excel (Microsoft office 2016)
